# Peer review of "A Robot Floating Grinding and Rust Removal Approach Based on Composite Force-Position Fuzzy Control"

_sensors, 2025, doi:10.3390/s25072204_

Round 1

Reviewer 1 Report

Comments and Suggestions for Authors

The article considers the problem of removing rust from the surfaces of large-sized equipment using a robotic system. The authors propose a solution based on the use of a floating end effector with composite force and position control. The developed effector is equipped with a force sensor and a laser displacement sensor to adapt to curved surfaces without precise knowledge of their geometry. The control algorithm uses a fuzzy differential PID controller to improve the accuracy and adaptability of grinding force control. The relevance of the work is due to the fact that traditional rust removal methods have disadvantages such as high cost, energy consumption and environmental pollution. The robotic approach allows for efficient, accurate and environmentally friendly rust removal. The importance of the study lies in the proposed solution, which allows automating the process of rust removal from large-sized objects of complex shape, ensuring high productivity and processing quality. The experimental results show that the proposed approach provides a high rust removal rate (up to 99.73%) and stable grinding force control. A number of points in the article need to be revised.
1. The novelty of the study needs to be more clearly stated (lines 9-21). The abstract and introduction (lines 26-40) focus on solving an important problem, but it is not clearly stated what exactly about the proposed approach is new and superior to existing solutions. What are the specific limitations of existing methods that are addressed in this paper? What are the unique features of the developed floating effector and control algorithm that provide improved results? Comparisons with alternative approaches should be more specific and supported by references to relevant works. It is necessary to explain how this work advances the field of robotic rust removal. Having described these issues, it is worthwhile to slightly expand the Introduction section and add references to it.
2. Format the bibliography according to the MDPI standard.
3. Mathematical model of the system (lines 138-147). How were the equations of motion obtained? What assumptions were made when constructing the model? It is necessary to provide a more detailed description of the modeling process and justify the assumptions made. 4. Description of the fuzzy control algorithm. The rule table (lines 277-287) is presented without sufficient explanation of the logic behind the rule selection. It is necessary to explain how the input variables (force deviation and deviation change rate) are related to the controller output parameters (changes in PID controller coefficients).
5. The methodology also needs to clarify the following questions - What software was used? What modeling parameters were chosen?

6. What parameters were varied in the experiments? How were the grinding force and rust removal rate measured?

7. It is necessary to analyze the effect of grinding speed on rust removal quality. The article mentions that at a speed of 60 mm/s, rust removal quality decreases (lines 370-374). It is necessary to analyze the causes of this phenomenon in more detail and propose ways to eliminate it.
8. The section on modeling (lines 138-154) lacks information on model validation. Were the modeling results compared with experimental data? If yes, what were the results obtained?

Comments on the Quality of English Language

It is necessary to check the whole article for grammatical and stylistic errors. There are many typos and inaccuracies in the text, which make it difficult to perceive the information. It is recommended to engage a professional editor to proofread the text.
Examples
line 12 ‘ The end-effector employs a force sensor and a laser displacement sensor to measure the deviation between the robot's programmed path and the actual contour of the workpiece,’ (Replace ‘uses’ with ‘employs’ and add ‘programmed’ for clarification).
line 73 ‘In this paper, a floating grinding actuator design will be introduced, which only needs to use the robot's teach pendant to plan and run the robot, and at the same time, use the independent grinding’ The sentence is very long and confusing. There are a lot of unnecessary words.
and so on

Author Response

Comments 1: [Should 1. The novelty of the study needs to be more clearly stated (lines 9-21). The abstract and introduction (lines 26-40) focus on solving an important problem, but it is not clearly stated what exactly about the proposed approach is new and superior to existing solutions. What are the specific limitations of existing methods that are addressed in this paper? What are the unique features of the developed floating effector and control algorithm that provide improved results? Comparisons with alternative approaches should be more specific and supported by references to relevant works. It is necessary to explain how this work advances the field of robotic rust removal. Having described these issues, it is worthwhile to slightly expand the Introduction section and add references to it.]

Response 1: [Dear judging experts, thank you very much for your questions and suggestions. In this regard, I would like to make the following answers and modify the article.

First, as per your suggestion, the abstract (sections 9-30) and introduction (sections 35-51) of the article have been revised. Second, the specific limitations of existing methods are as follows:For large-scale equipment rust removal, traditional methods (chemical cleaning, flame, and laser) suffer from high energy consumption, complex operation, and narrow applicability. Sandblasting and high-pressure water jet technologies have high costs and cause environmental pollution. Existing robotic systems rely on pre-measured data, leading to low control precision, complex path planning, poor adaptability to complex surfaces, and other challenges that make them difficult to generalize.

The end-effector floating grinding actuator designed in this paper operates without requiring pre-measured workpiece geometry data. It uses force-position hybrid sensing for real-time compensation of surface curvature errors, overcoming the limitations of traditional methods that rely on offline programming. A fuzzy derivative-before PID algorithm is developed for grinding force control, demonstrating superior performance compared to conventional control approaches. Through a hybrid compensation strategy combining displacement pre-compensation and force control, continuous rust removal on complex surfaces is achieved, with a maximum rust removal rate of 99.73%.

Therefore, the proposed solution in this paper is superior to existing methods for large-scale equipment rust removal, providing a highly efficient, environmentally friendly, and high-precision automated solution for this application.] 

Comments 2: [Format the bibliography according to the MDPI standard.]

Response 2: [Dear judging experts, thank you for pointing out the mistakes. I have changed the manuscript according to the format required by MDPI. If there are still mistakes, please correct me.] 

Comments 3: [There  Mathematical model of the system (lines 138-147). How were the equations of motion obtained? What assumptions were made when constructing the model? It is necessary to provide a more detailed description of the modeling process and justify the assumptions made.]

Response 3: [ Dear evaluation experts, thank you very much for your question, and give the following answers. The kinematic equations in this paper are cited from Reference [22], which describes the dynamic modeling of the pneumatic system for the end-effector servo mechanism and control system design of the steel wire brush grinding robot previously developed by our research group. Based on the same principles, we designed and fabricated the actuator hardware with an identical system transfer function framework, while updating the hardware parameters.] 

Comments 4: [Description of the fuzzy control algorithm. The rule table (lines 277-287) is presented without sufficient explanation of the logic behind the rule selection. It is necessary to explain how the input variables (force deviation and deviation change rate) are related to the controller output parameters (changes in PID controller coefficients). ]

Response 4: [Dear judging experts, thank you very much for your question. In this regard, I would like to make the following answers and modify the article. Regarding the principles followed in establishing the rule table, the correlation between input variables and output parameters was supplemented in Article (295-303).

The rule design principle remains that when the output error is large, error elimination should be prioritized; when the output error is small, control stability should be emphasized. For example, when the force deviation e is large (e.g., NB/NM), the system is in a transient state far from the target. In this case, rapid deviation elimination should be prioritized by setting a large ΔKP to enhance response and a small ΔKi to avoid overshoot. When the force deviation e is small (e.g., ZO/PS) and the rate of change of deviation ec is low, the system approaches steady-state. Here, a small ΔKP suppresses oscillation while a large ΔKi reduces steady-state error. When the deviation change rate ec is large (e.g., PB/NB), indicating rapid system fluctuations, differential effect is introduced to pre-suppress trend deviations and enhance stability

Simulation and experimental results demonstrate that at a speed of 40mm/s, the force fluctuation of fuzzy differential prior PID is reduced by 37% compared to traditional PID (Tables 3-4), and the rust removal rate reaches 97.08% (Table 5). Thank you for your guidance. We will further refine the description of the fuzzy control algorithm to enhance the interpretability of the paper.] 

Comments 5: [The methodology also needs to clarify the following questions - What software was used? What modeling parameters were chosen?]

Response 5: [Dear judging experts, thank you very much for your question. In this regard, I would like to make the following answers and modify the article. 

The software tools used in this study are described as follows: The simulation analysis was conducted using MATLAB/Simulink to build the Power control algorithm model (Fig. 8), with a simulation step size set to 1 ms. For image processing, the identification of rusted areas and calculation of removal rates were performed using custom-developed image processing software based on OpenCV. The detailed steps can be found in lines 116-123 of the article and (see Figs. 2 and 12 for details). The control system was developed in a Python environment, implementing control algorithms on a real-time operating system with display capabilities. The modeling parameters for the pneumatic system were derived from Ref. [22], as shown in Table 1 of the article.] 

Comments 6: [ What parameters were varied in the experiments? How were the grinding force and rust removal rate measured?]

Response 6: [Dear evaluation experts, thank you very much for your question. In this regard, the following is an explanation of the experimental design and measurement method in this paper: 

According to the article (lines 360-368), the experimental parameters were variables based on two control algorithms: fuzzy differential leading PID and traditional PID. Grinding and rust removal experiments were conducted at different advancing speeds of 20 mm/s, 40 mm/s, and 60 mm/s respectively. The fixed parameters included a target grinding force of 20 N, a motor speed of 4000 rpm, and a planned path point count of 5.

The grinding force was output by a pressure sensor as an analog voltage signal (0–5 V range), which was converted before entering motion control. The rust removal rate quantification method (as described in lines 114-121 of the article) utilizes OpenCV image processing technology to identify rust and statistically calculate the removal rate. Randomly collect images after derusting using an electron microscope, convert them to HSV color space, set HSV thresholds to segment rust areas, generate a mask, perform grayscale conversion, apply the mask to set rust regions to pure black and non-rust regions to white, and finally calculate the pixel ratio of rust areas in the entire image.] 

Comments 7: [It is necessary to analyze the effect of grinding speed on rust removal quality. The article mentions that at a speed of 60 mm/s, rust removal quality decreases (lines 370-374). It is necessary to analyze the causes of this phenomenon in more detail and propose ways to eliminate it. ]

Response 7: [Thank you for pointing this out. I agree with this comment, and make the following answers and modify the article. 

   Regarding the issue of reduced rust removal quality under the 60 mm/s feed speed condition (supplementary modification to lines 394-398 of the article), in-depth analysis reveals that the primary causes are: 1. Pneumatic actuator response limitations: The response speed of the pneumatic actuator may not keep up with the curvature change rate of the path, leading to height compensation delays and affecting contact force stability. Future designs could implement improvements such as high-speed proportional valves or more rigid mechanical structures. 2. Path planning impacts: Although the paper mentions using 5 path points, under high-speed operation, errors in path interpolation may be amplified, causing larger deviations between the actual grinding path and the ideal path, thus reducing rust removal uniformity. The improvement solution involves setting an appropriate number of path points according to the complexity of the workpiece shape.] 

Comments 8: [ The section on modeling (lines 138-154) lacks information on model validation. Were the modeling results compared with experimental data? If yes, what were the results obtained?]

Response 8: [Dear evaluation experts, thank you very much for your question, and give the following answers to this. 

Your comment points out the deficiency in model validation within the modeling section of the paper. This study does not directly compare model predictions with experimental data. Currently, the modeling section primarily verifies system stability through frequency-domain analysis (Bode plots and Nyquist plots), which provides theoretical support for the model's rationality. Although explicit validation was not conducted, simulation results (Fig. 9) demonstrate that system responses align with expectations, and the fuzzy differential PID controller exhibits superior force control performance compared to traditional PID in experiments. To further enhance model credibility, we plan to conduct system identification experiments in future work to optimize the model parameters using measured data.] 

4. Response to Comments on the Quality of English Language

Point 1: Point one: you need to check the grammar and stylistic errors of the whole article. There are many typos in the text, which are inaccurate and difficult to perceive information.

Response 1: Thank you for pointing this out. Because my poor editing and language errors have caused you trouble, I have checked and revised the full text to fix the wrong split words and grammatical errors. Make changes to lines 12 and 73 of the text.

5. Additional clarifications

[No additional clarification.]

Reviewer 2 Report

Comments and Suggestions for Authors

his paper proposes a new method of robot floating grinding based on force-position fuzzy control. The application to rust removal in large-scale equipment is industrially relevant, and the fusion of fuzzy differential prior PID control with force-position compensation presents a novel technical approach. 
1. The author mentions the floating grinding actuator will control both the force and the height of the position for grinding and descaling. So during the grinding process, is the speed of fuzzy pid resolution sufficient?  And after defuzzying, could the pneumatic system respose in time?2.Since the fuzzy rules are based on the grinding force deviation and the rate of change of the grinding force deviation, could the rules meet the processing requirements of different materials?

3.In line 337, the experimental platform is power off, could you provide a powered working figure with the Beckhoff controller?

4.In order to give some clear comparison of the algorithms, could you please also give some block diagram or parameters of the traditional PID, to show the differences clearly to readers, and as well to show your innovation to readers?

5.The paper mentions test results but doesn't compare them with existing methods. How does their fuzzy PID compare to other advanced control strategies? Also, the methodology is a bit vague. 

6.Please improve the English style.

Comments on the Quality of English Language

The English could be improved to more clearly express the research.

Author Response

Comments 1: [1. The author mentions the floating grinding actuator will control both the force and the height of the position for grinding and descaling. So during the grinding process, is the speed of fuzzy pid resolution sufficient?  And after defuzzying, could the pneumatic system respose in time?]

Response 1: [Dear judging experts, thank you very much for your question. In this regard, I would like to make the following answers and make amendments to the article. 

The fuzzy differential priority PID controller put forward in the paper can stabilize and track the target force within just 0.21 seconds during the step - response process, as shown in Figure 9a. In lines 173 - 181 of the article, the stability of the pneumatic system is confirmed. To boost the response speed, the paper makes use of a displacement sensor to predict contour changes, as described in Section 3, Equation 6. By doing so, it can pre-compensate for height errors in advance, which helps reduce the instantaneous pressure adjustment of the pneumatic system. Moreover, the design of the fuzzy rule table in Table 2 gives priority to eliminating large errors, thus further optimizing the dynamic response. Nevertheless, in the grinding and rust - removal experiments, when the speed reaches 60 mm/s, the compensation ability of the system weakens, resulting in poor rust - removal effects. In the future, to enhance the performance under extreme working conditions, improvements can be made by upgrading pneumatic components such as high - speed proportional valves or optimizing the control cycle. ] 

Comments 2: [2.Since the fuzzy rules are based on the grinding force deviation and the rate of change of the grinding force deviation, could the rules meet the processing requirements of different materials?]

Response 2: [Dear judging experts, thank you very much for your question. The following is an answer to your question. 

The current material processing in this paper is only applied to Q235 curved steel plates, without direct testing on multiple materials. However, the algorithm framework provides a foundation for multi-material expansion. Fuzzy control does not rely on precise models and can adapt to nonlinearity and uncertainty through adjusting membership functions (Figure 7) and fuzzy rules. For example, for different material hardness or friction characteristics, control requirements can be adapted by optimizing the rule table (e.g., increasing the weight of "negative large" responses for high-hardness materials). The paper's experiments achieved a 99.73% rust removal rate on curved steel plates (Table 5), demonstrating excellent performance in single-material scenarios. Therefore, the core control framework (compound force-position fuzzy control) exhibits general applicability.] 

Comments 3: [In line 337, the experimental platform is power off, could you provide a powered working figure with the Beckhoff controller?.]

Response 3: [Thank dear judging experts, thank you very much for your question. Here is our response:

In the early stage of the experiment, the Beckhoff controller and TwinCAT software system were used for development and verification. However, for practical promotion and cost considerations, a prototype based on Linux-based ARM host computer and Python environment was adopted in the later stage. The corresponding modifications were made in Lines 350-354 of the article.] 

Comments 4: [In order to give some clear comparison of the algorithms, could you please also give some block diagram or parameters of the traditional PID, to show the differences clearly to readers, and as well to show your innovation to readers? ]

Response 4: [Dear judging experts, thank you very much for your question. The following is an answer to your question. In the third section of the article, Figure 9 (simulation comparison chart) clearly presents the design of the proposed algorithm and the traditional PID algorithm. Additionally, lines 332-333 of the article supplement the description of control parameters for the traditional PID algorithm, while lines 230-233 include relevant explanations about the traditional PID algorithm. ] 

Comments 5: [The paper mentions test results but doesn't compare them with existing methods. How does their fuzzy PID compare to other advanced control strategies? Also, the methodology is a bit vague.]

Response 5: [Dear judging experts, thank you very much for your question. In this regard, I would like to make the following answers and make amendments to the article.

The paper currently only compares the traditional PID control and does not involve other advanced methods. This is primarily due to the compatibility limitations of the experimental platform and the implementation complexity of certain algorithms. The fuzzy derivative-priority PID proposed in this paper outperforms traditional PID in terms of force fluctuation and response speed through dynamic parameter adjustment and displacement compensation (e.g., 37% reduction in fluctuation at 40mm/s). Future research could include comparative experiments with other advanced methods to further validate the algorithm's advantages under extreme operating conditions.] 

Comments 6: [ Please improve the English style.]

Response 6: [Thank you for pointing that out. Because my editing and language errors have caused you trouble, I have checked and revised the full text to fix the wrong split words and grammatical errors.] 

5. Additional clarifications

[No additional clarification.]

Reviewer 3 Report

Comments and Suggestions for Authors

Through floating end-effector design, intelligent control algorithm development, experimental validation, and industrial compatibility optimization, this article achieves technological breakthroughs in robotic grinding and rust removal, combining academic innovation with engineering practicality. However, there are some issues that need to be improved.

  1. The words "descaling" and "derusting" are used interchangeably (e.g., abstract and introduction). Suggestion: Unified as "rust removal" or "descaling". And repeatedly: "the the errors" (p. 7). Fixed: Changed to "the errors".
  2. Figure 9(a)(b): Axis labels are missing units (e.g., time "seconds", force "Newtons"). And Table 2 (fuzzy rules): Membership functions (e.g., Gaussian function parameters) are not explicitly defined.
  3. The "maximum membership averaging method" (defuzzification) is not well explained. Suggestion: Supplemental math formulas or pseudocode.
  4. The grinding force fluctuation (±2.34N at 60 mm/s) is mentioned, but there is no correlation between surface roughness or tool wear. Recommendation: Supplemental SEM image or roughness (Ra value) analysis.

Author Response

Comments 1: [1. The words "descaling" and "derusting" are used interchangeably (e.g., abstract and introduction). Suggestion: Unified as "rust removal" or "descaling". And repeatedly: "the the errors" (p. 7). Fixed: Changed to "the errors".]

Response 1: [Dear reviewers, thank you very much for your questions. The following responses are provided:

I sincerely apologize for the terminology error in the article. I have thoroughly rechecked the document and corrected the terminology error related to "rust removal". Regarding the error on page 7, I have rectified it and also reviewed other sections to ensure consistency and accuracy. ] 

Comments 2: [2.Figure 9(a)(b): Axis labels are missing units (e.g., time "seconds", force "Newtons"). And Table 2 (fuzzy rules): Membership functions (e.g., Gaussian function parameters) are not explicitly defined.]

Response 2: [Dear Dear reviewers, thank you very much for your questions. The following responses are provided:

In the revised version, we have added "time/s (i.e., time in seconds)" to the horizontal axis of Figure 9 (a) and "simulate force /N (i.e., simulated force in Newtons)" to the vertical axis. For Figure 9 (b), we have supplemented "time/s" on the horizontal axis and "simulate force /N" on the vertical axis.

Thank you for the expert’s careful corrections! In lines 281-287 of the article, specific parameters for the membership functions are provided. The input variables e and ec adopt a combination of Gaussian functions and triangular membership functions: the Gaussian functions use parameters μ = -3, σ = 0.5 (corresponding to the NB region) and μ = 3, σ = 0.5 (corresponding to the PB region), while the triangular membership functions are defined with vertices positioned at (-3, -2, -1) for the NM region, (-2, -1, 0) for the NS region, (-1, 0, 1) for the ZO region, and similarly defined positions for regions spanning from NM to PM .] 

Comments 3: [The "maximum membership averaging method" (defuzzification) is not well explained. Suggestion: Supplemental math formulas or pseudocode]

Response 3: [Thank Dear reviewers, thank you very much for your questions. The following responses are provided:

Regarding the issue of insufficient explanation for the "mean of maximum method" (defuzzification) raised by the reviewers, supplementary description and mathematical expression (Equation 15) have been added in Lines 307-316 of the article.] 

Comments 4: [ The grinding force fluctuation (±2.34N at 60 mm/s) is mentioned, but there is no correlation between surface roughness or tool wear. Recommendation: Supplemental SEM image or roughness (Ra value) analysis ]

Response 4: [ Dear reviewers, thank you very much for your questions. Here are the following responses:

You mentioned that the analysis of surface roughness and tool wear is indeed a shortcoming in the current research. This study focuses on verifying the improvement effect of the composite force-position fuzzy control strategy on the stability of grinding force and the rust removal rate. Therefore, the experimental design mainly revolves around force fluctuations and the rust removal rate. Regarding the correlation between surface quality and tool wear, we have made the following supplements in Lines 408-416 of the article:

The TR100 metal surface roughness tester was used to measure Ra values of ground areas under different feed speeds. Experimental samples after grinding at various speeds were measured, with the best Ra mean value reaching up to 2.9±0.8 μm.] 

5. Additional clarifications

[No additional clarification.]

Round 2

Reviewer 1 Report

Comments and Suggestions for Authors

The reviewers have done a significant job of finalizing the article, taking into account most of the reviewer's comments. However, some responses and revisions require more detail and specificity.
1. For the current version of the article, it is desirable to add at least a qualitative comparison of the modeling results with experimental data (for example, to note that the general trends coincide).
2. Fuzzy control - Add specific examples illustrating the operation of the rules in various situations.
3. In response to reviewer #1's comment, specific numbers are given based on the test results; it is desirable to transfer them to the text of the article to add credibility.

Author Response

Comments 1: [For the current version of the article, it is desirable to add at least a qualitative comparison of the modeling results with experimental data (for example, to note that the general trends coincide)]

Response 1: [ Dear Reviewer, thank you very much for your question. In response, I would like to provide the following answer:

In the revised manuscript, we have added a qualitative analysis comparing model simulation and experimental data in lines 442-454.

 Both simulation and experimental results validate the effectiveness of the fuzzy differential prior PID algorithm. Simulation results demonstrate that the fuzzy differential prior PID reduces the settling time by 26.92% in step response compared to traditional PID, with significantly reduced overshoot and superior stability in sinusoidal tracking. The simulated trends align well with experimental findings: under gradually increasing forward velocities, the force fluctuation range and average error of fuzzy differential prior PID control are notably better than those of traditional PID. Specifically at 40 mm/s, the fuzzy differential prior PID maintains force fluctuations within ±1.34N and a rust removal rate of 97.08%, while traditional PID exhibits ±2.14N fluctuations, verifying the robustness advantage predicted by the model. Overall, the consistent trends between simulation and experimental data confirm that the theoretical advantages of fuzzy differential prior PID in dynamic response and stability can effectively translate into practical performance improvements."] 

Comments 2: [Fuzzy control - Add specific examples illustrating the operation of the rules in various situations.]

Response 2: [Dear Reviewer, thank you very much for your question. In response, I would like to provide the following answer:

We have supplemented the following specific examples in lines 224-331 of Section 4 and elaborated on the operation of fuzzy rules under different operating conditions in lines 295-305. 

For example when the actuator initially contacts the workpiece with actual grinding force significantly less than target force, grinding force deviation e is determined as NB (e=-3) and deviation rate ec as NB. To accelerate response and reduce error, the fuzzy rule table outputs Δkp=PB (+3), Δki=NB (-3). Output variables Δkp and Δki directly take domain values due to only a single maximum point at this moment. Mapped to actual Δkp adjustment +30%, Δki adjustment −30%. The previous tuning obtained parameters kp0 and ki0 as 400 and 11. According to equation 16 in the article, the current final control parameters can be calculated as kp = 400 + 400 × 30% = 520 and ki = 11 − 11 × 30% = 7.7. Simulation results show adjustment time of fuzzy differential leading PID is reduced by 26.92% compared to traditional PID in step response, validating the effectiveness of parameter adjustment.

Examples of fuzzy rule table outputs under different operating conditions are also presented. During stable grinding and rust removal, when e is small (ZO) but ec is PB, reduce proportional gain to suppress oscillations and enhance integral action to eliminate residual errors with rule output Δkp=NM, Δki=PM to maintain force stability; during high-speed grinding under high-frequency interference where force deviation e is ZO and sudden curvature changes cause deviation rate to surge to PB, triggering Δkp=NM to avoid oscillations and Δki=PM enhancement to compensate for delays to suppress high-frequency disturbances.] 

Comments 3: [ In response to reviewer #1's comment, specific numbers are given based on the test results; it is desirable to transfer them to the text of the article to add credibility.]

Response 3: [  Dear Reviewer, thank you very much for your question. In response, I would like to provide the following answer:

We have reorganized the test results and directly incorporated critical data into the main text, with specific revisions as follows: In the grinding and rust removal experiment section, key data has been embedded in lines 395-403 of the manuscript. Additionally, critical data related to the measured Ra values has been integrated into lines 424-427.] 
